# Real-world use of disease-modifying therapy in persons with multiple sclerosis aged 55 and over

**DOI:** 10.1136/bmjno-2025-001108

**Published:** 2025-09-23

**Authors:** Mina Stanikić, Anke Salmen, Christian P Kamm, Patrick Roth, Pasquale Calabrese, Chiara Zecca, Claudio Gobbi, Claudia Baum, Benjamin Victor Ineichen, Viktor von Wyl, on behalf of Swiss Multiple Sclerosis Registry, Mina Stanikic

**Affiliations:** 1 Department of Epidemiology, University of Zurich Institute of Epidemiology Biostatistics and Prevention, Zürich, Switzerland; 2 University of Zurich Institute for Implementation Science in Health Care, Zürich, Switzerland; 3 Department of Neurology, Ruhr University Bochum, Bochum, NRW, Germany; 4 Department of Neurology, Inselspital Universitatsspital Bern, Bern, BE, Switzerland; 5 Neurocentre, Luzerner Kantonsspital, Lucerne, LU, Switzerland; 6 Department of Neurology, University Hospital Zurich, Zürich, ZH, Switzerland; 7 Deptartment of Neurology, University Hospital Basel, Basel, BS, Switzerland; 8 Neuropsychology and Behavioral Neurology Unit, University of Basel, Basel, BS, Switzerland; 9 Neurocenter of Southern Switzerland, Ospedale Regionale di Lugano, Lugano, Switzerland; 10 Universita della Svizzera italiana Facolta di scienze biomediche, Lugano, TI, Switzerland; 11 Rehabilitation Clinic Zihlschlacht, Zihlschlacht-Sitterdorf, Switzerland; 12 Department of Clinical Research, University of Bern, Bern, Switzerland

**Keywords:** MULTIPLE SCLEROSIS, EPIDEMIOLOGY

## Abstract

**Background:**

As the average age of multiple sclerosis (MS) population rises globally, unclear guidelines on disease-modifying therapy (DMT) use in older persons with MS (pwMS) contribute to increased variability in clinical practice. The factors driving DMT utilisation in this population are not well understood. We explored DMT utilisation patterns in pwMS aged 55 and older enrolled in the Swiss MS Registry (SMSR), a nationwide observational study with voluntary participation.

**Methods:**

We conducted an exploratory analysis using data from SMSR participants who had reported DMT status in the most recent follow-up survey and at least once within the previous 3 years. Participants were categorised and compared by current and past DMT use: *No DMT* (no use), *Stopped* (prior use), *Continued* (same DMT), *Switcher* (changed DMT) and *New* (initiated DMT). Log-binomial regression identified factors associated with non-use, grouping participants as No DMT (*No DMT*, *Stopped*) and DMT (*Continued, Switcher, New*).

**Results:**

Among 378 participants (mean age 63.2±6.7 years), 206 (54.5%) reported DMT use: 176 (46.6%) continued the same DMT, 20 (5.3%) switched and 10 (2.6%) newly initiated DMT. Among non-users, 54 (14.3%) had stopped treatment, while the rest did not use DMT during the study period. In participants with regular neurological care, longer MS duration (relative risk (RR)=1.018, 95% CI 1.008 to 1.028) and older age (RR=1.016, 95% CI: 1.001 to 1.032) were associated with higher likelihood of DMT non-use, and participants with primary (RR=1.736, 95% CI: 1.175 to 2.565) and secondary progressive MS (RR=1.423, 95% CI: 1.023 to 1.981) were more likely not to use DMTs compared with relapsing-remitting MS. No significant associations were observed in participants without regular neurological follow-up.

**Conclusions:**

Despite unclear efficacy and safety, many older pwMS continue DMT use. Use is primarily associated with relapsing-remitting MS, while age and disease duration show only modest or no association.

**WHAT IS ALREADY KNOWN ON THIS TOPIC**

- While safety and efficacy of disease-modifying therapies (DMTs) in older adults with multiple sclerosis (MS) remain uncertain, some studies suggest that discontinuation may be advisable beyond a certain age. In the absence of clear guidelines, clinical practice varies.

**WHAT THIS STUDY ADDS**

- We found minimal differences between older adults who continued or discontinued DMTs. MS duration and age were either modestly associated with higher likelihood of DMT non-use, or not at all, depending on the regularity of neurological care. This suggests that treatment decisions in older adults may be driven more by clinical indications and regulatory approvals than by chronological age or disease duration.

**HOW THIS STUDY MIGHT AFFECT RESEARCH, PRACTICE OR POLICY**

- Our findings highlight the lack of a decisive factor - beyond disease phenotype - guiding DMT use in older adults, reflecting the absence of a unified clinical consensus. This underscores the need for further research and clearer treatment guidelines in this population.

## Introduction

 Multiple sclerosis (MS) is shifting from a disease predominantly associated with young adulthood to one that increasingly affects older populations, with a growing proportion of persons with MS (pwMS) over the age of 50.^1^ This demographic change necessitates the refinement of disease-modifying therapy (DMT) strategies, as their risk-benefit profile may become less favourable in older individuals. Age-related changes, including reduced MS inflammatory activity, impaired repair mechanisms, physiological neurodegeneration^2^ and an elevated risk of malignancies, cardiovascular diseases and infections in older pwMS,^3^ complicate treatment decisions and may alter both the efficacy and safety profile of DMTs.

While meta-analyses show that DMT efficacy declines with age,^4 5^ these studies lack data on pwMS over the age of 55, as more than 90% of clinical trials leading to DMT approval have excluded this age group.^6^ Recent real-world evidence has begun to address this gap, with a large multicentre study demonstrating that ocrelizumab significantly outperformed interferon and glatiramer acetate in reducing relapses among individuals over the age of 60.^7^ Additional real-world data suggest that DMTs may confer benefits in older pwMS with active disease, including shorter hospital stays and fewer emergency room visits.^8^

However, these potential benefits must be weighed against safety concerns. While short-term use of high-efficacy DMTs in pwMS over 50 may be safe,^9^ long-term safety remains uncertain, with evidence suggesting an associated age-dependent risk of infections^10^ and malignancies.^11^ Furthermore, comorbidities may contraindicate DMT initiation^12^ or negatively impact adherence, tolerability and effectiveness.^13^ Finally, some studies indicate that discontinuing DMTs in older pwMS with stable disease, particularly those receiving lower-efficacy therapies, does not necessarily lead to clinical or radiological worsening,^14 15^ or may be associated with a low risk of new imaging activity of unclear clinical or long-term significance.^16^

Ultimately, limited data on DMT safety and efficacy in older pwMS has resulted in an absence of clear treatment guidelines, particularly regarding the appropriate timing of therapy discontinuation,^17 18^ likely leading to significant variability in clinical practice.

This exploratory study aims to assess real-world patterns of DMT use among older pwMS in Switzerland. Specifically, we seek to determine the proportion of pwMS aged 55 and older who are currently receiving DMTs, assess the continuity of their treatment, identify factors associated with DMT use and determine the most frequently used DMTs in this demographic.

## Methods

### Data source and study population

This study used self-reported data from the Swiss MS Registry (SMSR), an observational study of pwMS aged 18 and older who live or receive treatment in Switzerland. Participation is voluntary and requires signed informed consent and confirmation of an MS or clinically isolated syndrome (CIS) diagnosis. The SMSR collects socio-demographic, health and MS-related data every 6 months via online and paper questionnaires. Further details are available elsewhere.^19 20^

For this study, we primarily used data on DMTs and other MS-related information from the most recent follow-up survey at the time of analysis, released in October 2022, with data collection ending on 1 September 2023. To construct a recent history of DMT use, we also included data from the two preceding follow-up surveys. Socio-demographic information, such as education and citizenship, was sourced from the baseline survey completed at SMSR enrolment.

Inclusion criteria required participants to be 55 years or older at the time of the latest follow-up survey, have available data on age, sex and MS type, and report DMT use in both the latest follow-up survey and at least one of the two preceding surveys. Participants not meeting these criteria were excluded.

### Outcomes of interest

The primary outcome of interest in this study was DMT use. In all SMSR surveys, participants reported their DMT use over the past 6 months through a multiple-choice question listing both trade and generic drug names. They could select multiple options if applicable.

For descriptive analysis, participants were categorised into five predefined groups based on their DMT use across the study period of up to 3 years, spanning from the most recent follow-up survey (late 2023) to up to two prior surveys (late 2021–early 2022 and late 2020–early 2021). ‘Current’ use was defined using data from the most recent follow-up survey, while ‘past’ use incorporated data from one or both of the preceding surveys, depending on data availability. Based on this information, participants were categorised as follows: *No DMT* (no DMT use reported at any time during the study period), *Stopped* (DMT use within the study period but not currently), *Continued* (same DMT reported at least twice over the study period, including currently), *Switcher* (current use of a different DMT than previously reported within the study period) and *New* (current use with no past use reported during the study period). These categories reflect DMT use within the defined study period and do not capture use prior to it. A similar classification method has been successfully applied in a previous SMSR study.^21^ For regression models, we dichotomized the outcome into No DMT (combining *No DMT* and *Stopped*) and DMT (combining *Continued, Switcher* and *New*), that is, we utilized the DMT status from the most recent survey.

Additionally, to identify the most commonly used or discontinued DMTs, we created dichotomous (yes/no) variables for each DMT listed in the multiple-choice response options.

### Variables of interest

For descriptive analysis and variable selection in regression models, we considered self-reported sociodemographic, health-related and MS-specific factors.

The sociodemographic information was extracted from the SMSR baseline questionnaire and included: age (continuous), sex (binary; female or male), Swiss citizenship (binary; yes or no) and highest level of education (categorical; university or applied university degree, higher professional education, and mandatory schooling, high school and internship).

Health-related information included presence of comorbidities such as heart disease, type 2 diabetes, hypertension, depression and cancer (binary; yes or no) reported at any time from enrolment in the SMSR onwards, regular visits to a neurologist (binary; yes or no), use of complementary medicine (acupuncture, osteopathy, traditional Chinese medicine, homeopathy and naturopathy, binary; yes or no) and use of cannabis-based products (categorical; use on own initiative, use in consultation with a health professional or no use). These data were primarily sourced from the latest follow-up survey and supplemented with data from the two preceding follow-up surveys when missing.

Finally, MS-related variables included current MS type, MS duration, disability level, presence of MS symptoms (binary; yes or no) and relapses in the past 6 months (binary; yes or no), all sourced from the latest follow-up survey. MS type was assessed using a multiple-choice question with response options for relapsing-remitting MS (RRMS), secondary progressive MS (SPMS), primary progressive MS (PPMS), CIS and transition between RRMS and SPMS. Self-reported MS type was reviewed for consistency across SMSR questionnaires, participant comments and clinical records, which were available for approximately 8% of all SMSR participants. In cases of inconsistencies, participants were contacted for clarification. MS duration (continuous) was measured in years from symptom onset to questionnaire completion. Disability level was assessed using the Self-Reported Disability Status Scale, a validated Expanded Disability Status Scale (EDSS) proxy based on three mobility-related questions that classify individuals into EDSS-equivalent categories: ≤3.5, 4–6.5 and ≥7.^22^

### Analysis

Sociodemographic, MS-related and health-related characteristics were analysed descriptively and reported as percentages, means or medians to compare participants across the five DMT use groups.

To assess associations between various factors and DMT non-use cross-sectionally, we applied univariable and multivariable regression models. Since the outcome was common among the study participants, log-binomial regression was used instead of logistic regression, as it is more appropriate for modelling frequent outcomes.^23^ The multivariable model included sex, age and MS type as predefined predictors, with additional variables added sequentially and retained if the Akaike Information Criterion decreased by at least two units. Based on the same criterion and clinical rationale regarding treatment eligibility and DMT access, we tested interactions between MS type and age, MS type and MS duration, and between regular neurologist visits and both age and MS duration. Interaction terms retained in the model were further examined through subgroup analyses to assess effect modification. Model selection was performed using complete cases. Collinearity was assessed using df-adjusted generalised variance inflation factors (GVIFs). Variables with GVIF values greater than 5 were excluded from the model; otherwise, all variables were retained in the final model. We then used multiple imputation to generate and analyse 15 imputed datasets under the assumption that data were missing at random. Regression estimates were reported as relative risks (RRs) with 95% CIs for both the complete case and the imputed analysis.

To predict the probability of not using a DMT across different MS types, we applied the multivariable model as described above to the complete cases or to any subgroups identified through the interaction analyses. Additionally, a natural spline transformation for MS duration and age was included to capture potential non-linear effects. Predictions were generated using a dataset covering the range of MS duration and age observed in the study sample, holding other variables constant at their means (for continuous variables) or modes (for categorical variables). The predicted probabilities from both models—linear and spline-transformed—were then calculated and plotted against MS duration and age for each MS type.

All analyses were carried out using R (V.4.2.2)^24^ and packages lbm, splines and mice.

## Results

### Sample description

**Figure 1:**
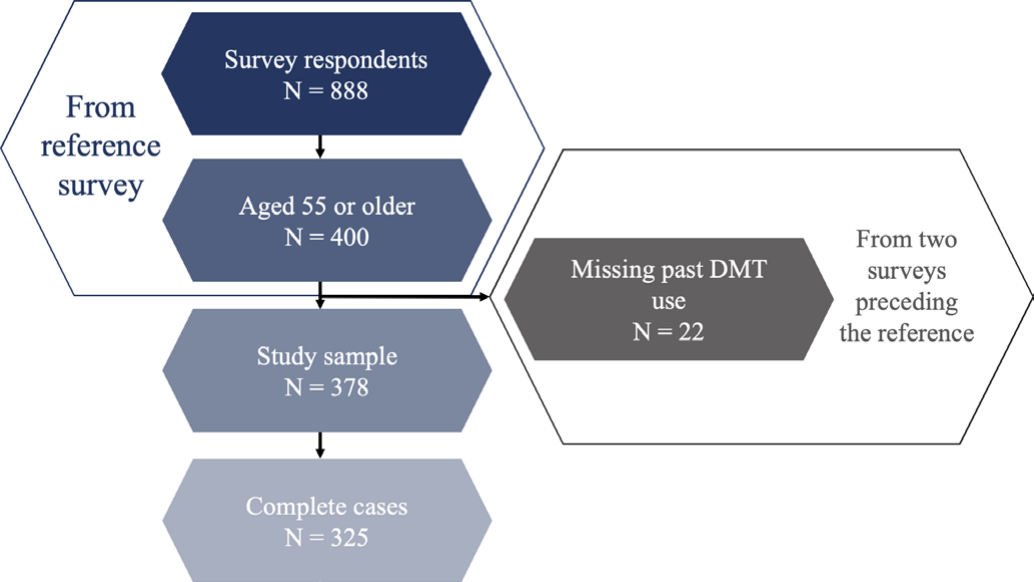
Study design flowchart. DMT, disease-modifying therapy.

A total of 888 SMSR participants completed the latest follow-up survey, with a response rate of 39.0%. Of these, 400 were aged 55 or older. However, 22 participants were excluded due to missing data on DMT use from prior surveys, leaving a final sample of 378. Of these, 278 (73.5%) had past DMT use constructed using data from both preceding surveys, while 100 (26.5%) had data from only one survey; among them, 65 (17.2%) had data only from the earlier preceding survey. For regression analyses, a subsample of complete cases (N=325) was used, including only participants with data available for all variables of interest (figure 1).

**Figure 2:**
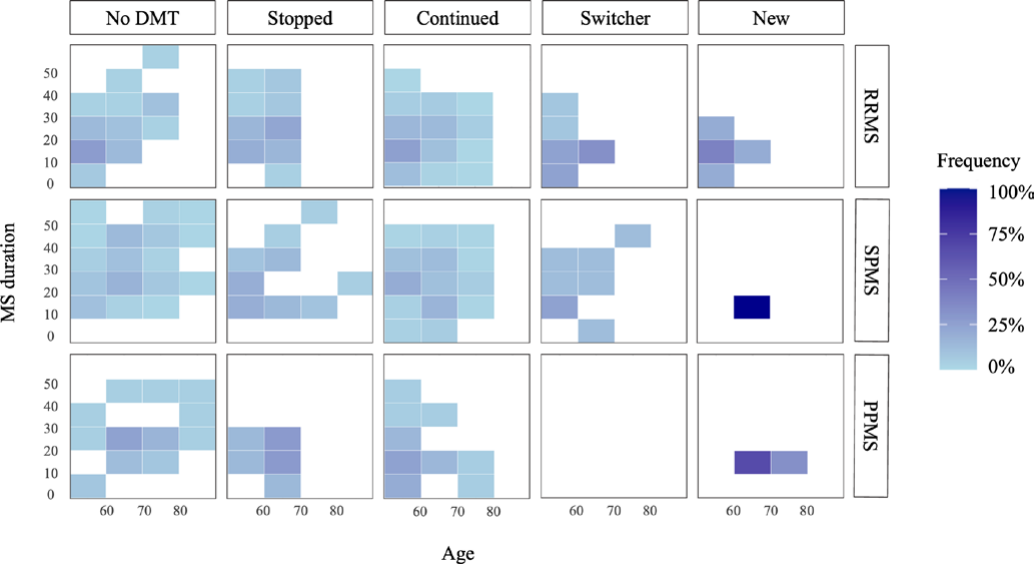
Distribution of participants across DMT use groups, stratified by MS duration and age categories. DMT, disease-modifying therapy; MS, multiple sclerosis; PPMS, primary progressive MS; RRMS, relapsing-remitting MS; SPMS, secondary progressive MS.

A total of 206 participants (54.5%) reported using DMTs. Of these, 176 (46.6%) continued with the same DMT (‘Continued’), 20 (5.3%) switched medications (‘Switcher’) and 10 (2.6%) newly initiated DMTs (‘New’) in the 3-year study period. Among those not using a DMT, 54 (14.3%) had stopped therapy in the latest follow-up survey (‘Stopped’), while the remainder had not used any DMT throughout the study period (‘No DMT’). For eight participants (7.07%) in the ‘No DMT’ group, the SMSR records suggest they have never used a DMT. Figure 2 shows the distribution of participants across DMT use groups, stratified by 10-year categories of MS duration and age, with frequencies calculated using the total number of participants in each DMT use/MS type group as a denominator.

Participants in the ‘No DMT’ group were more often male, older, had longer MS duration and had more frequently reported having SPMS compared with other groups. Additionally, they more commonly reported not seeing a neurologist, using cannabis-based products and using complementary therapies. The characteristics of the other groups were largely similar (table 1).

**Table 1:** Comparison of participants grouped by DMT use: sociodemographic, health-related and MS-related characteristics

Characteristic	No DMT	Stopped	Continued	Switcher	New	Overall
	(n=118)	(n=54)	(n=176)	(n=20)	(n=10)	(N=378)
**Sex, N (%**)						
Female	80 (67.8)	42 (77.8)	125 (71.0)	16 (80.0)	8 (80.0)	271 (71.7)
Male	38 (32.2)	12 (22.2)	51 (29.0)	4 (20.0)	2 (20.0)	107 (28.3)
**Age in years**						
Mean (SD)	66.2 (7.9)	62.9 (6.6)	61.6 (5.3)	60.4 (4.9)	62.1 (4.9)	63.2 (6.7)
Median (IQR)	65.0 (60.0–71.0)	62.0 (57.0–67.0)	60.0 (57.0–64.0)	58.5 (57.0–63.0)	61.5 (60.0–62.0)	62.0 (58.0–67.0)
**Swiss citizenship, N (%**)						
No	7 (5.9)	3 (5.6)	14 (8.0)	0 (0)	1 (10.0)	25 (6.6)
Yes	110 (93.2)	50 (92.6)	161 (91.5)	20 (100)	9 (90.0)	350 (92.6)
Missing	1 (0.8)	1 (1.9)	1 (0.6)	0 (0)	0 (0)	3 (0.8)
**Geographical region, N (%**)						
German-speaking	81 (68.6)	42 (77.8)	143 (81.3)	18 (90.0)	5 (50.0)	289 (76.5)
French-speaking	28 (23.7)	9 (16.7)	24 (13.6)	1 (5.0)	3 (30.0)	65 (17.2)
Italian-speaking	2 (1.7)	3 (5.6)	4 (2.3)	0 (0)	2 (20.0)	11 (2.9)
Romansh-speaking	0 (0)	0 (0)	2 (1.1)	1 (5.0)	0 (0)	3 (0.8)
Missing	7 (5.9)	0 (0)	3 (1.7)	0 (0)	0 (0)	10 (2.6)
**Highest education level, N (%**)						
Mandatory, high school or apprenticeship	69 (58.5)	36 (66.7)	102 (58.0)	11 (55.0)	5 (50.0)	223 (59.0)
Higher professional education	13 (11.0)	6 (11.1)	22 (12.5)	2 (10.0)	2 (20.0)	45 (11.9)
University or applied university	29 (24.6)	12 (22.2)	41 (23.3)	5 (25.0)	2 (20.0)	89 (23.5)
Missing	7 (5.9)	0 (0)	11 (6.3)	2 (10.0)	1 (10.0)	21 (5.6)
**MS duration in years**						
Mean (SD)	28.8 (11.9)	24.5 (11.0)	21.1 (9.81)	19.9 (10.6)	17.3 (4.67)	23.8 (11.2)
Median (IQR)	26.0 (20.0–38.0)	22.0 (16.0–30.0)	20.0 (14.0–27.0)	18.0 (15.3–26.5)	17.5 (17.0–18.0)	22.0 (16.0–31.0)
Missing, N (%)	1 (0.8)	1 (1.9)	7 (4.0)	0 (0)	0 (0)	9 (2.4)
**MS type, N (%**)						
CIS	1 (0.8)	0 (0)	0 (0)	0 (0)	1 (10.0)	2 (0.5)
PPMS	24 (20.3)	7 (13.0)	20 (11.4)	0 (0)	3 (30.0)	54 (14.3)
RRMS	28 (23.7)	26 (48.1)	101 (57.4)	12 (60.0)	4 (40.0)	171 (45.2)
SPMS	60 (50.8)	18 (33.3)	47 (26.7)	7 (35.0)	2 (20.0)	134 (35.4)
Transition	5 (4.2)	3 (5.6)	8 (4.5)	1 (5.0)	0 (0)	17 (4.5)
**EDSS proxy, N (%**)						
≤3.5	49 (41.5)	21 (38.9)	97 (55.1)	10 (50.0)	5 (50.0)	182 (48.1)
4–6.5	38 (32.2)	23 (42.6)	60 (34.1)	9 (45.0)	5 (50.0)	135 (35.7)
≥7	28 (23.7)	10 (18.5)	19 (10.8)	1 (5.0)	0 (0)	58 (15.3)
Missing	3 (2.5)	0 (0)	0 (0)	0 (0)	0 (0)	3 (0.8)
**MS symptoms in the last 6 months, N (%**)						
No	28 (23.7)	17 (31.5)	55 (31.3)	5 (25.0)	2 (20.0)	107 (28.3)
Yes	89 (75.4)	36 (66.7)	121 (68.8)	15 (75.0)	8 (80.0)	269 (71.2)
Missing	1 (0.8)	1 (1.9)	0 (0)	0 (0)	0 (0)	2 (0.5)
**Relapses in the last 6 months, N (%**)						
No	107 (90.7)	49 (90.7)	158 (89.8)	18 (90.0)	8 (80.0)	340 (89.9)
Yes	5 (4.2)	4 (7.4)	8 (4.5)	2 (10.0)	2 (20.0)	21 (5.6)
Missing	6 (5.1)	1 (1.9)	10 (5.7)	0 (0)	0 (0)	17 (4.5)
**Use of complementary medicine, N (%**)						
No	84 (71.2)	47 (87.0)	148 (84.1)	18 (90.0)	7 (70.0)	304 (80.4)
Yes	34 (28.8)	7 (13.0)	28 (15.9)	2 (10.0)	3 (30.0)	74 (19.6)
**Use of cannabis-based products, N (%**)						
No	82 (69.5)	44 (81.5)	136 (77.3)	16 (80.0)	6 (60.0)	284 (75.1)
Yes, medical consultation	17 (14.4)	4 (7.4)	21 (11.9)	2 (10.0)	2 (20.0)	46 (12.2)
Yes, own initiative	16 (13.6)	5 (9.3)	14 (8.0)	2 (10.0)	2 (20.0)	39 (10.3)
Missing	3 (2.5)	1 (1.9)	5 (2.8)	0 (0)	0 (0)	9 (2.4)
**Comorbidities, N (%**)						
No	83 (70.3)	35 (64.8)	128 (72.7)	15 (75.0)	5 (50.0)	266 (70.4)
Yes	34 (28.8)	19 (35.2)	48 (27.3)	5 (25.0)	5 (50.0)	111 (29.4)
Missing	1 (0.8)	0 (0)	0 (0)	0 (0)	0 (0)	1 (0.3)
**Regular visits to a neurologist, N (%**)						
No	38 (32.2)	4 (7.4)	3 (1.7)	2 (10.0)	0 (0)	47 (12.4)
Yes	80 (67.8)	50 (92.6)	173 (98.3)	18 (90.0)	10 (100)	331 (87.6)

CIS, clinically isolated syndrome; DMT, disease modifying therapy; EDSS, Expanded Disability Status Scale; MS, multiple sclerosis; PPMS, primary progressive MS; RRMS, relapsing-remitting MS; SPMS, secondary progressive MS.

### Use of DMT agents

**Figure 3:**
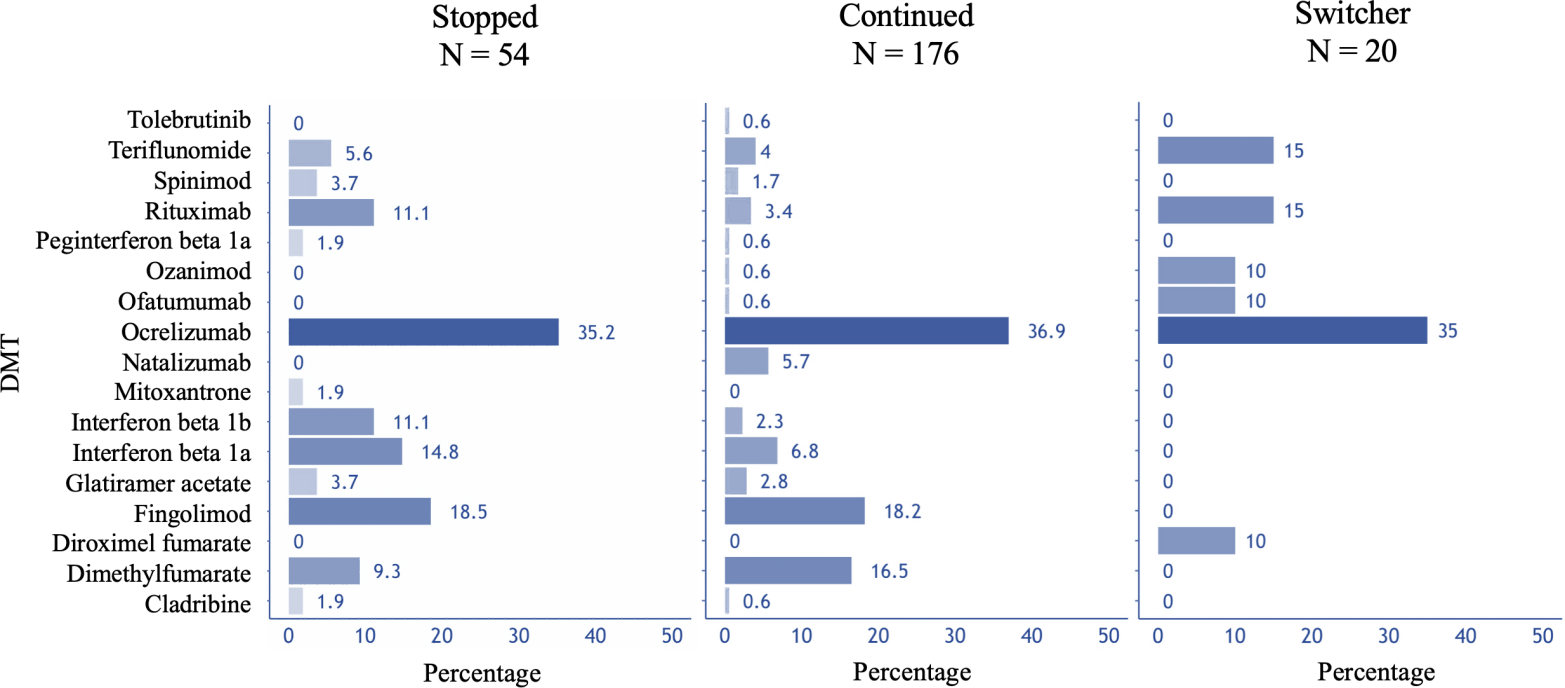
Frequency of use of DMT agents in groups ‘Stopped’, ‘Continued’ and ‘Switcher’. DMT, disease-modifying therapy.

Ocrelizumab was the most frequently used DMT across all groups that reported DMT use. In the ‘Continued’ group, 65 participants (36.9%) used ocrelizumab, followed by fingolimod (n=32, 18.2%) and dimethyl fumarate (n=29, 16.5%). In the ‘Switcher’ group, ocrelizumab was the most frequently switched-to medication (n=7, 35%). It was also the most frequently discontinued, with 19 out of 54 participants (35.2%) reporting having stopped its use (figure 3). In the ‘New’ group, 4 out of 10 participants used ocrelizumab, 1 used rituximab, 3 used dimethyl fumarate, 1 used diroximel fumarate and 1 used fingolimod.

### Factors associated with the use of DMTs

In the univariable regression analysis, older age, longer MS duration, progressive MS types and higher EDSS proxy were associated with a higher likelihood of not using a DMT. Additionally, French-speaking participants were more likely to forgo DMT use than German-speaking participants, while regular neurologist visits were associated with a lower likelihood of not using a DMT (table 2).

**Table 2:** Univariable and multivariable log binomial regression derived risk ratios (RR) and 95% CIs

Characteristic	Univariable analysis Complete case analysis N=310 RR (95% CI)	Multivariable analysis	
		Complete case analysis N=310 RR (95% CI)	Full sample analysis N=378 RR (95% CI)
**Sex**			
Female	Ref.	Ref.	Ref.
Male	0.977 (0.745 to 1.281)	1.028 (0.977 to 1.080)	1.037 (0.971 to 1.107)
**Age**	**1.036 (1.030 to 1.041**)	1 (1 to 1)	1.001 (0.997 to 1.005)
**Swiss citizenship**			
No	Ref.		
Yes	0.988 (0.567 to 1.721)		
**Language region**			
German	Ref.		
French	**1.375 (1.064 to 1.776**)		
Italian	0.927 (0.428 to 2.008)		
**Highest education level**			
Primary school, high school or apprenticeship	Ref.		
Higher professional education	0.834 (0.555 to 1.253)		
University or applied university	0.933 (0.698 to 1.247)		
**MS duration**	**1.024 (1.020 to 1.028**)	1.002 (0.998 to 1.007)	1.002 (0.999 to 1.005)
**MS subtype**			
RRMS	Ref.	Ref.	Ref.
PPMS	**1.868 (1.323 to 2.636**)	**1.448 (1.104 to 1.900**)	**1.43 (1.119 to 1.829**)
SPMS	**1.848 (1.387 to 2.461**)	**1.400 (1.059 to 1.850**)	**1.393 (1.086 to 1.786**)
**EDSS proxy**			
≤3.5	Ref.		
4–6.5	1.269 (0.961 to 1.676)		
≥7	**1.722 (1.292 to 2.294**)		
**MS symptoms in the last 6 months**			
No	Ref.		
Yes	1.121 (0.853 to 1.473)		
**Relapses in the last 6 months**			
No	Ref.		
Yes	0.961 (0.565 to 1.635)		
**Use of complementary medicine**			
No	Ref.		
Yes	1.231 (0.932 to 1.625)		
**Use of cannabis-based products**			
No	Ref.		
Yes, medical consultation	0.876 (0.571 to 1.344)		
Yes, own initiative	1.231 (0.881 to 1.721)		
**Comorbidity**			
No	Ref.		
Yes	1.109 (0.864 to 1.426)		
**Regular visits to a neurologist**			
No	Ref.	Ref.	Ref.
Yes	**0.448 (0.373 to 0.537**)	**0.291 (0.208 to 0.405**)	**0.276 (0.201 to 0.380**)
**MS duration × regular visits to a neurologist**			
No	–	Ref.	Ref.
Yes	–	**1.022 (1.016 to 1.027**)	**1.023 (1.017 to 1.028**)

EDSS, Expanded Disability Status Scale; MS, multiple sclerosis; PPMS, primary progressive MS; RRMS, relapsing-remitting MS; SPMS, secondary progressive MS.

The final multivariable model, with no evidence of problematic collinearity (GVIF values range: 1.05–1.39), included sex, age, MS duration, MS type, regular neurologist visits and interaction between regular neurologist visits and MS duration (table 2). Given this statistically significant interaction, regression estimates were reported separately for participants with (N=331 imputed; N=272 complete case) and without regular neurological care (N=47 imputed; N=38 complete case) in table 3. In the regular care subgroup, each additional year of MS duration was associated with a 1.6–1.8% higher risk of DMT non-use, and each additional year of age with a 1.2–1.6% higher risk, although the latter reached statistical significance only in the imputed analysis. Participants with progressive MS were more likely to be non-users than those with RRMS, with the risk being greater for PPMS than for SPMS. In contrast, no statistically significant associations were found in the non-regular care subgroup (table 3).

**Table 3:** Univariable and multivariable log binomial regression derived risk ratios (RR) and 95% CIs from subgroup analysis by neurological care status

	In regular neurological care		Not in regular neurological care	
**Characteristic**	**Complete case analysis** **N=272** **RR (95% CI**)	**Imputed analysis** **N=331** **RR (95% CI**)	**Complete case analysis** **N=38** **RR (95% CI**)	**Imputed analysis** **N=47** **RR (95% CI**)
**Sex**				
Female	Ref.	Ref.	Ref.	Ref.
Male	0.985 (0.727 to 1.333)	1.016 (0.843 to 1.223)	1.024 (0.978 to 1.073)	1.042 (0.963 to 1.127)
**Age**	1.012 (0.99 to 1.035)	**1.016 (1.001 to 1.032**)	1 (1 to 1)	1 (0.999 to 1)
**MS subtype**				
RRMS	Ref.	Ref.	Ref.	Ref.
PPMS	**1.758 (1.144 to 2.701**)	**1.736 (1.175 to 2.565**)	1.306 (0.954 to 1.789)	1.261 (0.899 to 1.768)
SPMS	**1.505 (1.044 to 2.17**)	**1.423 (1.023 to 1.981**)	1.268 (0.908 to 1.770)	1.200 (0.824 to 1.748)
**MS duration**	**1.016 (1.006 to 1.026**)	**1.018 (1.008 to 1.028**)	1.002 (0.998 to 1.006)	1.003 (0.997 to 1.008)

MS, multiple sclerosis; PPMS, primary progressive MS; RRMS, relapsing-remitting MS; SPMS, secondary progressive MS.

### Predicted probability of DMT use

**Figure 4:**
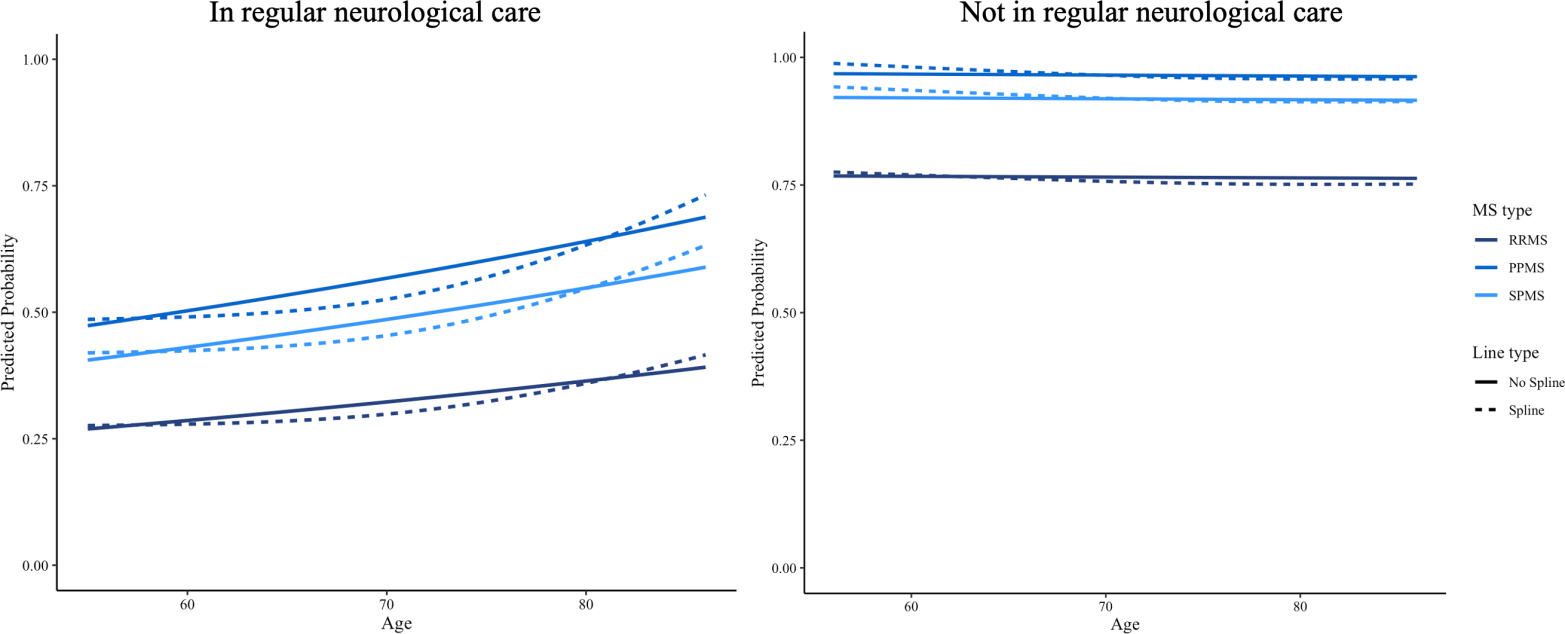
Predicted probability of DMT non-use by age in RRMS, PPMS and SPMS, stratified by neurological care. DMT, disease-modifying therapy; MS, multiple sclerosis; PPMS, primary progressive MS; RRMS, relapsing-remitting MS; SPMS, secondary progressive MS.

Figures4  5 show the predicted probability of not using a DMT as a function of age and MS duration across three MS types among participants with and without regular neurological care. While the spline-transformed model reveals some fluctuations, the overall trend remains similar over time, with RRMS consistently showing a lower probability of not using a DMT compared with PPMS and SPMS in both subgroups of participants.

**Figure 5:**
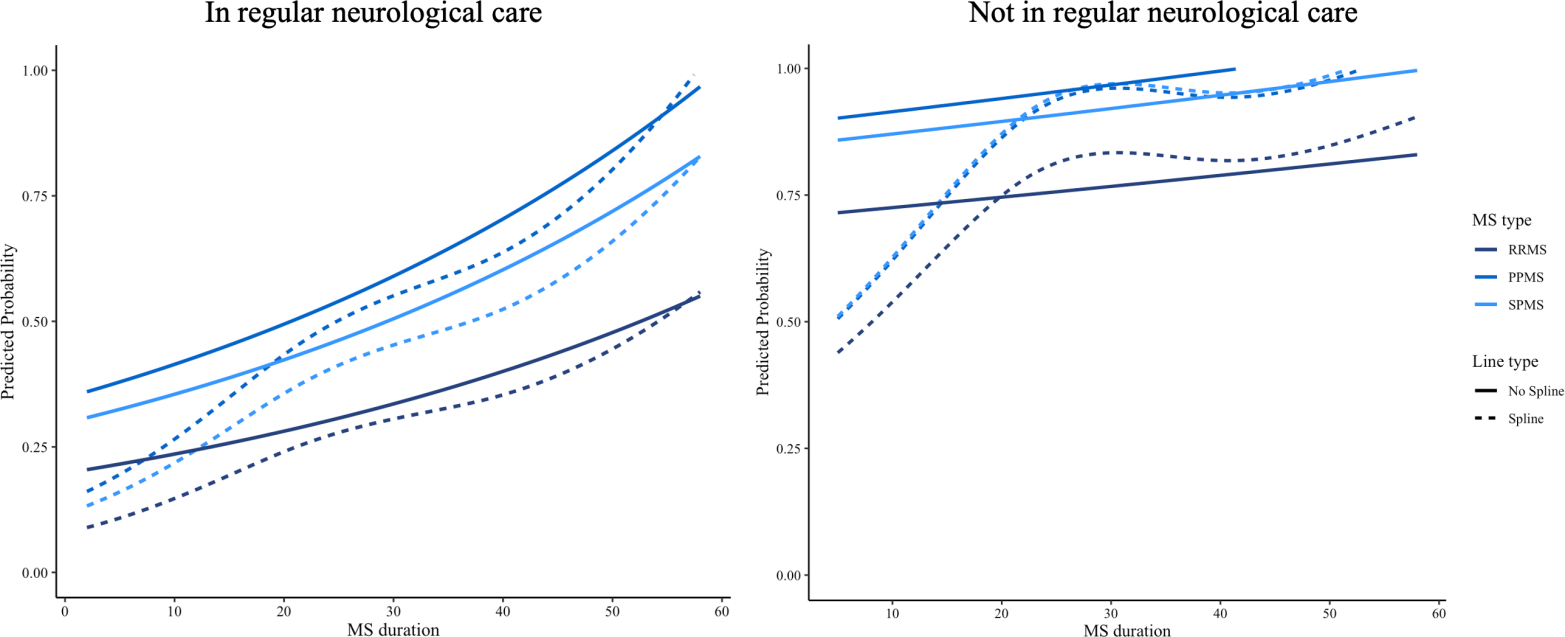
Predicted probability of DMT non-use by MS duration in RRMS, PPMS and SPMS, stratified by neurological care. DMT, disease-modifying therapy; MS, multiple sclerosis; PPMS, primary progressive MS; RRMS, relapsing-remitting MS; SPMS, secondary progressive MS.

## Discussion

We analysed DMT use among 378 SMSR participants aged 55 and older, categorising them based on current and past DMT use. Just over half reported using DMTs, predominantly continuing the same therapy over the 3-year study period, while some had switched or initiated DMTs within this timeframe. Among non-users, one-third had discontinued DMTs, while two-thirds had not used them in the past 3 years. Non-users were generally older, had longer MS duration, greater disability and more frequently reported progressive MS. In the regular care subgroup, longer MS duration and, to a lesser extent, older age were associated with a higher likelihood of DMT non-use and progressive MS—particularly PPMS—was linked to greater non-use compared with RRMS. No significant associations were identified in the non-regular care subgroup. Ocrelizumab was the most commonly used and most frequently discontinued DMT.

Previous studies have reported varying DMT use rates among older pwMS, depending on the study setting, timing, health insurance coverage and data sources. In the North American Registry for Care and Research in MS, 40.1% of pwMS over 60 reported DMT use in 2018.^25^ In contrast, a US study analysing federal electronic medical records found only 18.9% of those over 60 received DMTs between 2016 and 2019.^26^ A German MS registry study using clinical data reported DMT use at 60.9% for those aged 55–64 and 42.6% for those 65+ as of 2023.^27^ Our study, based on highly accurate self-reported data,^28^ found 54.5% of pwMS aged 55 and over used DMTs in 2023. These findings align with previous studies, considering differences in participant age, recent DMT approvals, accessible healthcare in Switzerland and varying treatment guidelines.

Although Switzerland offers a range of DMTs comparable to the European market, differences in regulatory approvals between neighbouring countries and independent Swiss cost–benefit assessments can influence first-line and second-line treatment choices.^29^ Ocrelizumab was the most frequently reported DMT in our study, consistent with findings from the German MS registry among older pwMS.^27^ The widespread use of high-efficacy DMTs among both continuous users and switchers in our study is somewhat unexpected, given concerns about increased infection and neoplasm risks in older pwMS on these therapies.^10 11^ However, factors such as a disease duration of less than 5 years and presence of relapses in some participants, along with the fact that more than half of those with PPMS were either using or had recently discontinued treatment—presumably ocrelizumab—may have contributed to this finding.

In Switzerland, DMT reimbursement requires an annual report from a board-certified neurologist, meaning that patients without regular follow-up are intrinsically less likely to receive therapy or have it reimbursed. Accordingly, the observed strong association between absence of regular neurological visits and DMT non-use was anticipated, as was the observed effect modification by regularity of neurological care. Within the regular care subgroup, longer disease duration and older age were statistically significantly, though modestly, associated with DMT non-use. This is consistent with prior observations that older pwMS generally exhibit lower DMT utilisation than younger individuals,^27 30^ although it only partially aligns with previous evidence reporting no association between disease duration and persistence on injectable DMTs^31^; the modest effects of age and disease duration are likely influenced by adjustment for MS type. In the same subgroup, progressive MS was associated with lower likelihood of DMT use, reflecting the until-recently limited availability of therapies with proven efficacy in preventing disability progression in PPMS and SPMS, and ongoing uncertainty about whether current treatments can modify relapse-independent progression in SPMS.^32^ In contrast, no significant associations were observed in the non-regular care subgroup, which had a small sample size and likely reflects a heterogeneous population of patients who might have exhausted therapeutic options, those perceiving limited benefit from therapy or individuals encountering disease-related (eg, physical or cognitive) or structural and logistical barriers to specialist access.^33^

MS treatment discontinuations typically result from lack of efficacy, intolerance or side effects.^34^ While our surveys included questions on reasons for discontinuation, most participants who stopped treatment did not provide an answer, leaving too few data points for analysis. However, participants who discontinued treatment did not differ considerably from those who continued, with median ages and MS durations varying by only 2 years. This is consistent with a previous study that found no significant differences between those who continued and those who discontinued treatment in pwMS over the age of 60.^35^ The most notable difference in our study was that those who discontinued treatment reported comorbidities more frequently. While physical comorbidities are not generally associated with DMT persistence in the broader MS population,^36^ they play a key role in neurologists’ treatment decisions for older pwMS, alongside disease activity and age.^37^

Recent discontinuation trials have demonstrated lower rates of inflammatory disease recurrence after DMT cessation in older pwMS compared with younger patients,^16 38^ suggesting that discontinuation may be safe in carefully selected individuals with long-term clinical and radiological stability. This is particularly relevant for those receiving low-efficacy or B-cell–depleting therapies,^16 39^ such as ocrelizumab, the most commonly used DMT in our cohort, suggesting that a proportion of older pwMS may be overtreated.^40^ Nevertheless, further clinical trials and real-world studies are needed to inform discontinuation practices and assess long-term outcomes following treatment cessation.

### Strengths and limitations

Our study provides valuable real-world insights into DMT use among older pwMS, addressing gaps left by the absence of specific guidelines. We used nationwide self-reported DMT data with excellent validity and reliability across all ages.^28^ Additionally, our hybrid approach combined cross-sectional data with prior treatment patterns, including discontinuation and switches. However, there are limitations to consider. For 17.2% of participants, past DMT use was collected during the early phase of the COVID-19 pandemic (late 2020 to early 2021), a period marked by potentially altered prescribing patterns, which may have influenced their DMT grouping in the present analysis. Furthermore, we were unable to analyse the reasons for DMT discontinuation due to the limited number of available data points. This paucity of data may reflect the survey design, as participants were only asked to report reasons if the discontinuation had occurred within the preceding 6 months. Additionally, while MS type reporting in the SMSR is generally valid and reliable, its accuracy declines with age and among those with progressive MS,^28^ potentially introducing bias. To mitigate this, we used adjudicated MS type in our analyses, as described earlier. Lastly, Switzerland’s near-universal healthcare and high availability of MS medications may limit the generalisability of our findings to healthcare settings with different levels of access.

## Conclusions

In Switzerland, over half of pwMS aged 55 and above were using DMTs. Non-users were older, had longer MS durations and more often reported progressive MS. However, after adjusting for MS subtype, both age and MS duration showed a statistically significant, although modest in magnitude, association with DMT use among participants in regular neurological care, whereas neither was significantly associated with use among those without regular care. This suggests that age and MS duration may not be the primary factors driving DMT use decisions. Recent discontinuers were similar to continuing users in age and MS duration but had higher comorbidity rates. These findings highlight the complexities of DMT use and discontinuation in older pwMS, emphasising the need for more specific guidelines and further research to better understand the factors influencing treatment decisions.

## Data Availability

Data are available upon reasonable request.
